# Melkersson-Rosenthal syndrome: a rare variant of the monosymptomatic form

**DOI:** 10.3205/oc000191

**Published:** 2022-02-08

**Authors:** Carolina Tagliari Estacia, Aluisio Rosa Gameiro Filho, Izadora Bouzeid Estacia da Silveira, Rodrigo Rosa Gameiro, Anna Laura Salabert Della Barba

**Affiliations:** 1 Hospital Federal dos Servidores do Estado do Rio de Janeiro (HFSERJ), Rio de Janeiro, Brazil; 2 Universidade Federal de São Paulo – Escola Paulista de Medicina (UnifespEPM), São Paulo, Brazil; 3 Universidade Federal de Ciências da Saúde de Porto Alegre (UFCSPA), Porto Alegre, Brazil; 4 Pontifícia Universidade Católica do Paraná (PUCPR), Curitiba, Brazil

**Keywords:** Melkersson-Rosenthal syndrome, eyelid, eyelid edema, case reports

## Abstract

Melkersson-Rosenthal syndrome (MRS) is a rare condition without any known etiology. It is characterized by the triad facial paralysis, facial swelling, and the development of folds and furrows at the tongue (fissured tongue). This study aims to report a case of a 59-year-old patient complaining about asymmetric eyelid swelling that had started two years before, associated with pain and redness on her right eye, without repercussions on her visual acuity. The patient underwent a skin biopsy of the right eye’s lower eyelid, which was compatible with the monosymptomatic form of MRS. Consequently, five injections of triamcinolone were performed for the period of one year, with gradual and satisfactory improvement. One year after the end of the treatment, the patient returned with recurrence of the swelling, and therapy with triamcinolone associated with oral steroids was started. However, due to the lack of improvement, Tacrolimus ointment 0.03% was initiated. The patient evolved with an important and significant reduction of the eyelid edema, still being followed at the Hospital Federal dos Servidores do Estado do Rio de Janeiro.

## Introduction

Melkersson-Rosenthal syndrome (MRS) is a rare condition of unknown aetiology. Its classic oligosymptomatic form is characterized by the presence of orofacial edema, the development of folds and furrows at the tongue (fissured tongue), and recurrent peripheral facial paralysis being the first, the most frequent and characteristic symptom. Facial paralysis occurs in only 20–30% of the cases [1]. The monosymptomatic form is defined by the presence of periorbital swelling, without the other findings of the triad. This condition has no predilection for race or gender. Our aim is to report this rare syndrome in its monosymptomatic form, which is often misdiagnosed or underdiagnosed.

## Case description

**Figure 1:**
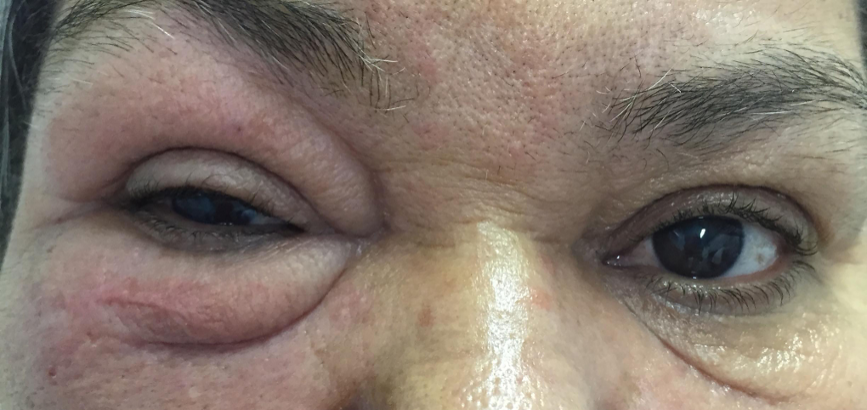
Eyelid edema at the right side

A 59-year-old female patient, resident of Rio de Janeiro, sought the ophthalmology ambulatory at the Hospital Federal dos Servidores do Estado do Rio de Janeiro, complaining about right eyelid edema, which had started two years before, associated with itching and pain (Figure 1 (Fig. 1)). She denied trauma or any previous eye surgery. She also referred systemic arterial hypertension and diabetes mellitus (non-insulin dependent).

At biomicroscopy, bilateral blepharitis associated with local hyperemia and soft eyelid edema at the right eye was noticed, without any other abnormalities. The patient’s best corrected visual acuity was 20/20 in both eyes. Intraocular pressure (IOP) was also normal, and fundoscopy revealed narrow arteriolar and pathological arteriovenous crossings, compatible to hypertensive retinopathy.

Treatment for blepharitis was initiated, and laboratory tests (blood count; coagulogram; biochemistry; renal, hepatic, and thyroid function tests) were requested, as well as allergic tests and abdominal ultrasound, all of them with normal results. Orbital computed tomography (OCT) was also performed, showing thickening and expansion of the soft tissues of both the upper and the lower right-eye eyelid, without orbital involvement. A biopsy of the lower eyelid skin of the right eye showed areas of edema of the dermis, ectasia of lymphatic vessels, capillary dilatation, and inflammatory infiltrate, compatible with the monosymptomatic form of Melkerson-Rosenthal syndrome.

**Figure 2:**
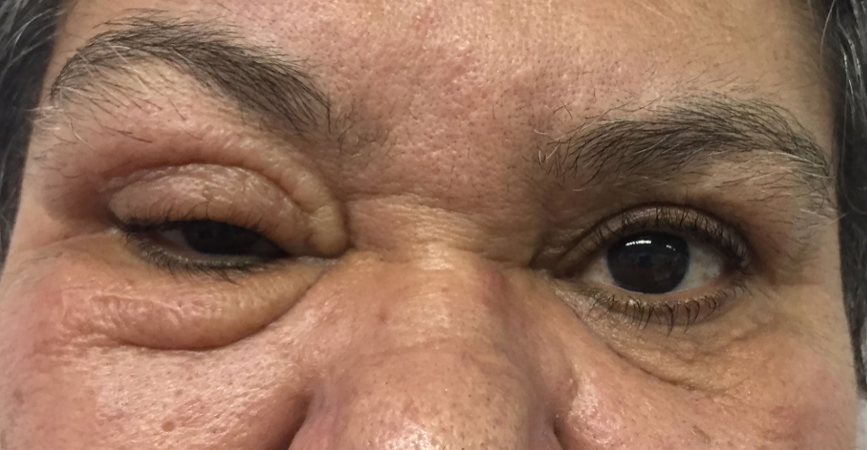
Recurrence of the eyelid edema on the right side

**Figure 3:**
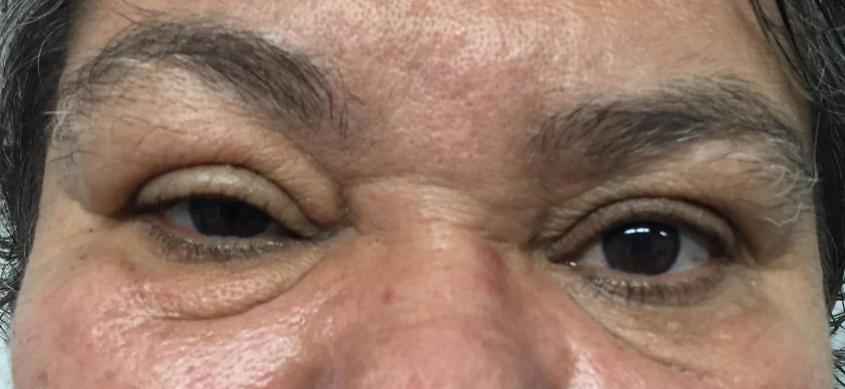
Aspect after treatment with tacrolimus; the patient is still being followed at our ambulatory.

Treatment with subcutaneous triamcinolone injections in the upper and lower eyelids of the right eye was proposed. A total of five injections were performed over the period of one year, with a gradual and satisfactory improvement of the condition, although incomplete. One year later, the patient returned with recurrence of the eyelid edema on the right eye (Figure 2 (Fig. 2)), and therapy with new injections of Triamcinolone was instituted quarterly, associated with oral steroids (Prednisone 40 mg for 5 days). Due to the insignificant improvement, Tacrolimus 0.03% ointment was initiated, with gradual improvement (Figure 3 (Fig. 3)). The patient has been under follow-up ever since 2016.

## Discussion

Melkersson-Rosenthal syndrome (MRS) is a rare granulomatous neuro-mucocutaneous condition [1] characterized by the triad facial paralysis, ipsilateral facial edema, and fissured tongue. In 1928, Melkersson described the paralysis and facial edema, and 3 years later, Rosenthal observed that those symptoms were presented in outbreaks, and were frequently associated with fissured tongue [2]. Nevertheless, it was only in 1949 when the term ‘Melkersson-Rosenthal syndrome’ was used for the first time, by Luscher [3], [4]. MRS is an uncommon condition, with an incidence of 0.08% in the general population. However, this incidence must be higher since cases are frequently underreported. It has no gender or racial predilection [5].

Even though having been described as early as almost a century ago, its etiology remains unknown. Some authors suggest associations with abnormalities of enervation; chronic infections (such as herpes) [6]; hypersensitivity to bacteria; Crohn’s disease; and sarcoidosis. Genetic predisposition has also been proposed: a mutation at the gene ATP1 (fatty acid transport protein) has been described in a Chinese family [7]. Subsequently, the heterozygous variant SLC27A1 (FATP1) has also been reported as responsible for MRS [8], albeit more studies are necessary to confirm those associations.

Regarding the symptoms, the classic triad is only described in 25–30% of cases [9]. Fissured tongue is always congenital [5]. Peripheral facial palsy can occur from months to years either before or after the facial swelling, and it can be partial or complete, unilateral or bilateral, and is sometimes clinically indistinguishable from Bell’s palsy. Last but not least, facial swelling is the most frequent finding, present in 86% of cases [5]. When it is located only on the lips, it is also known as Miescher’s cheilitis [6]. It usually has a chronic course, with remissions and relapses, and occasional spontaneous resolution. In our case, the patient presented with isolated eyelid edema, which is uncommon, painless, non-pitting, and has an insidious onset. Other ophthalmological findings associated with the syndrome include: retrobulbar neuritis, corneal opacities, keratoconjuntivitis sicca, and blepharochalase [1].

The diagnosis is usually clinical [10]. However, especially in our case, in which only periocular edema was present, other conditions must be excluded, such as tumors, pseudotumors, lymphoproliferative disease, thyroid ophthalmopathy, and blepharochalase. The use of CT scan or even nuclear magnetic resonance (NMR) can be helpful, showing thickening of the soft tissues, which is compatible with lymphocytes infiltration, edema, and dilatation of lymphatic vessels. A biopsy is a *sine qua non* procedure for patients with periorbital edema without apparent cause, and usually reveals granulomatous angeiitis with perivascular inflammatory cells. Granulomas can be seen both within and around the vessels or distributed in the variable dermal edema, or even on the orbiculares and elevator muscles. Albeit, it is important to highlight that the pathological findings are non-specific. Some patients can show granulomatous folliculitis, and some, like ours, show only perivascular lymphocytic infiltrates.

With regard to treatment, intralesional injections of steroids are efficient for resolution for both eyelids and mouth swelling [11], [12]. Systemic steroids are associated with a greater chance of remission. If treatment fails, immunosuppressants can be considered. The surgical approach, such as blepharoplasty or nerve decompression, has already been reported and can be used as a last option for complex and challenging cases. Unfortunately, to date, no single combination therapy leads to complete remission of MRS [13].

## Conclusion

Due to its rarity, MRS is usually ignored or misdiagnosed. As it does not have a single presentation, MRS should be considered in cases of uni- or bilateral periorbital edema, especially in recurrent cases. Biopsies should be performed routinely in all patients with eyelid edema of unknown etiology. A conservative approach is always recommended [14], with short-acting oral steroids appearing to be the basis of clinical treatment. In addition to that, Triamcinolone seems to be effective in case of orofacial edema, and immunosuppressants should be reserved for refractory/recurrent cases [15]. Treatment remains challenging.

It should be noted that the ophthalmologist must be familiar with the Melkersson-Rosenthal syndrome and with its possibility of presenting itself on oligo- or monosymptomatic forms.

## Notes

### Informed consent

Informed consent has been obtained from the patient for the publication of this case report.

### Authors’ ORCIDs

- Carolina Tagliari Estacia: 0000-0001-8186-4975
- Aluisio Rosa Gameiro Filho: 0000-0002-8787-0417
- Rodrigo Rosa Gameiro: 0000-0001-9600-6228

### Competing interests

The authors declare that they have no competing interests.
